# Developmental rewiring of EHMT1 function during human cortical neurogenesis

**DOI:** 10.64898/2026.10.09.757098

**Published:** 2026-10-12

**Authors:** Kritika Kasliwal, Kaushiki Chatterjee, Vittoria Dickinson Bocchi, Syed R. Mahmood, Christopher M Uyehara, William Marchese, Alejandra Laguillo-Diego, Emily Goetzler, Gabriel Theis, Andrew Minotti, Ryan Walsh, Alexander Polyzos, Eftychia Apostolou, Simone Sidoli, Lorenz Studer, Matthias Stadtfeld

## Abstract

Ubiquitously expressed chromatin regulators orchestrate cell-type-specific gene expression programs, yet how they acquire distinct regulatory functions during development remains fundamentally unresolved ^1,2^ . Using temporally controlled protein depletion during human cortical neurogenesis *in vitro* , we show that the autism-associated histone methyltransferase EHMT1 ^3,4^ is developmentally repurposed from epiblast to cortical neurons. Broadly expressed zinc finger proteins recruit EHMT1 to distinct cis-regulatory elements within changing chromatin landscapes associated with lineage-specific transcription factor activity, with extensive remodeling of EHMT1 enhancer occupancy during neural induction dependent on ZNF462. This genomic rewiring is accompanied by a fundamental shift from restraining premature developmental gene expression in pluripotent cells to supporting neuronal programs, with distinct transcriptional consequences of EHMT1 loss across neural progenitors and excitatory and inhibitory neuronal subtypes. Timed depletion and recovery experiments identify the neural progenitor stage as the onset of a developmental period during which EHMT1 is required for faithful neuronal maturation. Remarkably, EHMT1 can re-engage stage-specific targets after prolonged deficiency, allowing extensive transcriptional recovery with only limited molecular scarring. These findings reveal how ubiquitous epigenetic machinery acquires developmental specificity while demonstrating the resilience of developmental gene regulatory programs to prolonged disruption, with implications for neurodevelopmental disorders.

## Full Text Availability

The license terms selected by the author(s) for this preprint version do not permit archiving in PMC. The full text is available from the preprint server.

